# Flotillin-2 promotes nasopharyngeal carcinoma metastasis and is necessary for the epithelial-mesenchymal transition induced by transforming growth factor-β

**DOI:** 10.18632/oncotarget.3382

**Published:** 2015-04-07

**Authors:** Liang Zhao, Li Lin, Changqie Pan, Min Shi, Yulin Liao, Jianping Bin, Wangjun Liao

**Affiliations:** ^1^ Department of Oncology, Nanfang Hospital, Southern Medical University, Guangzhou Guangdong 510515, China; ^2^ Department of Cardiology, Nanfang Hospital, Southern Medical University, Guangzhou Guangdong 510515, China

**Keywords:** nasopharyngeal carcinoma, Flotillin-2, transforming growth factor-β, epithelial-mesenchymal transition, metastasis

## Abstract

Transforming growth factor-β (TGF-β) promotes cancer metastasis via the epithelial-mesenchymal transition (EMT) but the underlying mechanisms in nasopharyngeal carcinoma (NPC) remain unclear. Flotillin-2 (Flot2), a specialized lipid raft domain in cellular membrane, was reported to promote cancer metastasis. Recently, in neuropathy, it was also suggested that Flot2 was involved in Src activation, which is known as the downstream signal of TGF-β. Therefore, we intended to find out the relationship between Flot2 and TGF-β in the process of nasopharyngeal carcinoma (NPC) metastasis. In this study, we found that Flot2 expression level positively correlated with the cancer stage in NPC tissues. Elevated Flot2 in tumor tissue was an independent prognostic marker, and higher Flot2 expression level showed shorter overall survival time in 181 NPC patients. In NPC cells, silencing Flot2 reversed the metastatic effect induced by TGF-β. Moreover, TGF-β-induced Src phosphorylation was significantly inhibited by Flot2 knocking down. As the consequence of Flot2 inhibition, the expression of the epithelial biomarker E-cadherin was upregulated, while the mesenchymal marker vimentin and signaling transducer β-catenin was suppressed. In conclusions, Flot2 is an indispensable member for TGF-β signaling, which is essential for the EMT process in NPC metastasis. Suppressing Flot2 may be a novel way against TGF-β-induced EMT.

## INTRODUCTION

In epithelium originated cancer tissues, cancer cells are usually kept in epithelial polarity and connected by tight intercellular networks. When epithelial-mesenchymal transition (EMT) starts, cells gradually loss typical epithelial characteristics and gain mesenchymal features, which consequently facilitate cancer cells invasion and migration [1–5]. This phenomenon is regulated by series extracellular and intracellular signals [1–3]. Transforming growth factor-β (TGF-β) is an extracellular cytokine that known to promote tumor metastasis [1–11]. By activating some non-receptor tyrosine kinase, such as Src, TGF-β was reported to initiate EMT in various cancers [2, 3, 8]. However, the TGF-β-induced EMT and the key role of Src in this process have little been studied in nasopharyngeal carcinoma (NPC), which is an uncommon epithelial cancer in most regions but particularly more common in southeastern China [12].

The plain lipid raft is a cholesterol and sphingomyelin rich cytomembranous microdomain, which is known to connect with extracellular and intracellular signal transduction [13]. Flot2, an obbligato part of the plain lipid raft, mainly resides at the cytoplasmic side of cellular membrane and at the membranes of some intracellular vesicles [14, 15]. Recently, Flot2 was reported to interact with the cortical cytoskeleton and is required for a migration ability of neutrophils [16]. It was shown that overexpressing Flot2 transformed the nontumorigenic and nonmetastatic melanoma SB2 cells into highly tumorigenic and metastatic cells [17], while Flot2 deficiency leads to the deletion of plain raft and significant reduction of lung metastasis in breast cancer cells [18]. Moreover, Flot2 was reported as a lymphatic metastasis biomarker in NPC paraffin specimens [19]. However, the underlying mechanism for Flot2 promoting metastasis in cancer cells was still unclear. Recently, it was suggested that Flot2 kept the activity of Src and promoted axon regeneration in rat optic nerve cells [20]. These evidences indicate that Flot2 may interact with Src in the TGF-β intracellular signal transduction and promotes NPC cell metastasis.

Taken the aforementioned clues together, we hypothesized that Flot2 expression level may be associated with NPC metastasis in the TGF-β signal transduction. Accordingly, in this study, Flot2 expression levels were evaluated in NPC tissues from 181 followed NPC patients, and functional experiments were performed to confirm the pro-metastatic features of Flot2. Finally, the molecular role of Flot2 in regulating TGF-β associated EMT signaling was explored.

## RESULTS

### Flot2 is elevated in advanced NPC and associates with lymphatic and distant metastasis

**Figure 1:**
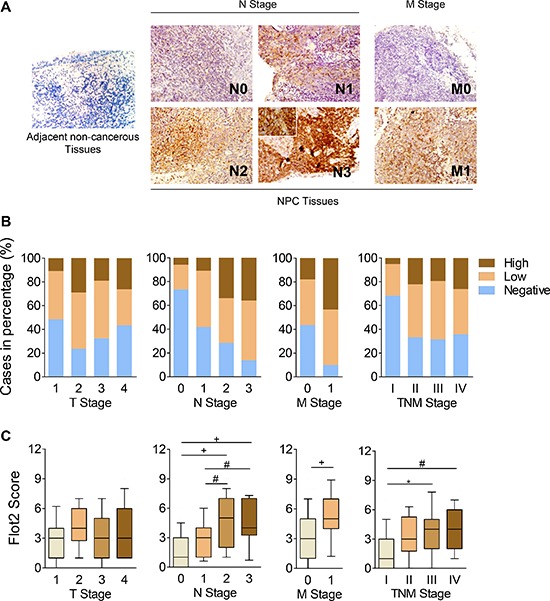
Flot2 is correlated with metastasis and indicates adverse prognosis in NPC patients **(A)** Representative IHC staining of Flot2 in adjacent noncancerous tissue and NPC tissue samples. Magnification 200 ×. **(B)** The frequency of negative, low and high Flot2 expression in NPC when categorized by TNM stages. **(C)** The average Flot2 staining scores of NPC tissue in different TNM stages.

To assay Flot2 expression, immunohistochemistry (IHC) staining was performed using NPC samples from 181 patients. We found that Flot2, which was predominant on cellular membrane, generally expressed higher in NPC tissues than their adjacent noncancerous tissues (Figure 1A). According to the Flot2 staining score, patients were divided into groups of negative (score 0–2), low (score 3–5) and high expressions (score > 5). There were 112 Flot2 positive patients, which took the majority (positives 61.9% vs. negatives 38.1%). Among them, 72 (39.8%) patients were defined as low expression, and 40 (22.1%) were as high expression. When categorizing the patients by tumor growth (T stages), lymphatic metastasis (N stages), distant metastasis (M stages) and overall TNM stages, we found that Flot2 high expression was more frequent in patients with more advanced N stage (*P* < 0.001), M stage (*P* < 0.001), and overall TNM stage (*P* = 0.006). However, no statistical significance was found in T stage (*P* = 0.337) (Figure 1B). The average Flot2 expression scores were also significantly higher in more advanced NPC of N, M and overall TNM stage, other than T stage (Figure 1C).

### Flot2 elevation indicates adverse prognosis for NPC patients

**Figure 2:**
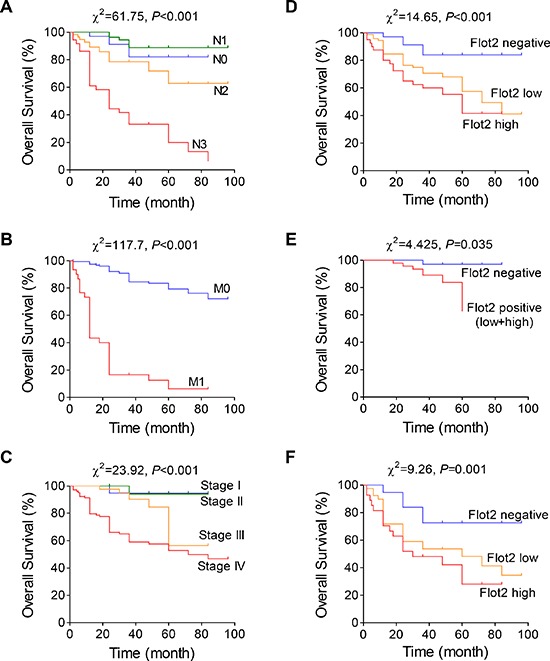
Flot2 indicates adverse prognosis in NPC patients **(A to C)** Kaplan-Meier analysis of NPC patient overall survival time by (A) lymphatic metastatic status, (B) distant metastatic status and (C) different TNM stages. **(D to F)** Higher Flot2 level indicates shorter overall survival in (D) all analyzed NPC patients, as well as in subgroups of (E) Stage I-III or (F) Stage IV patients.

Using COX proportional hazards regression model, the univariate relationships between tumor characteristics and patient survival were acquired. We found that Flot2 level, lymphatic metastasis (N stage), distant metastasis (M stage) status as well as overall TNM stage significantly influenced the overall survival of NPC patients (Table 1). However, no statistical significant influence was found by parameters of gender, age or T stage. Then, multivariate analysis of joint effect with the related parameters was performed. Likewise, only Flot2 level, N, M, and overall TNM stages are independent prognostic parameters for the survival outcomes of the NPC patients (Table 2). Next, Kaplan-Meier survival curve was plotted using aforementioned prognostic factors. The overall survival time was significantly shorter in patients with more advanced N (Figure 2A) and M (Figure 2B) stage. As the composite, patients with advanced TNM stage were also suffered with shorter survival time (Figure 2C). Most importantly, elevated Flot2 expression also showed shorter survival time in all patients (Figure 2D) as well as in subgroups of early (Stage I-III) (Figure 2E) and advanced (Stage IV) (Figure 2F) NPC. To be noted, because few patients of Stage I-III were of Flot2 high expression, we combined low and high expression as positive group (Figure 2E).

**Table 1:** The clinical features of NPC patients in this study

Variables	Cases	5-year OS (%)	*P* value
Gender			0.679
Female	68	48 (70.6%)	
Male	113	78 (69.0%)	
Age (year)			0.125
≤50	120	87 (72.5%)	
>50	61	39 (63.9%)	
T Stage			0.097
T1	37	31 (83.8%)	
T2	38	25 (65.7%)	
T3	38	26 (68.4%)	
T4	68	44 (64.7%)	
N Stage			<0.001*
N0	34	28 (82.3%)	
N1	55	49 (89.0%)	
N2	56	41 (73.2%)	
N3	36	8 (22.2%)	
M Stage			0.006*
M0	150	122 (81.3%)	
M1	31	4 (12.9%)	
Overall TNM Stage			<0.001*
I	19	18 (94.7%)	
II	17	16 (94.1%)	
III	40	35 (87.5%)	
IV	105	57 (54.2%)	
Flot2 Score			<0.001*
Negative	69	58 (84.0%)	
Low expression	72	46 (63.8%)	
High expression	40	22 (55.0%)	

**Table 2:** The multivariate analysis of the prognostic parameters in NPC patients

Variables	HR (95% CI)	*P* value
Age	1.011 (0.988–1.034)	0.367
Gender	1.265 (0.690–2.320)	0.447
T Stage	1.186 (0.894–1.573)	0.237
N Stage	1.763 (1.210–2.569)	0.003*
M Stage	5.681 (2.333–13.835)	0.003*
Overall TNM Stage	3.302 (2.215–4.923)	0.008*
Flot2 Score	1.502 (1.048–2.512)	0.007*

According to these clinical observations, Flot2 may play roles in the metastasis of NPC. However, whether Flot2 elevation was the consequence or the reason of NPC metastasis was unknown. Therefore, we used recombinant human TGF-β to induce cell motivation to see whether Flot2 was influenced.

### TGF-β promotes EMT via Src activation in NPC cells

**Figure 3:**
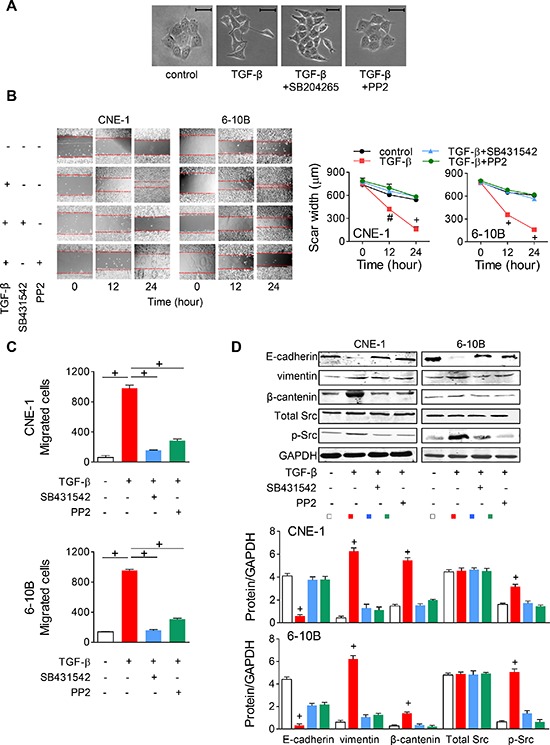
TGF-β promotes EMT via Src activation in NPC cells **(A)** TGF-β-induced NPC cell scattering and morphologic changes in CNE-1 cells were suppressed by SB431542 (TGF-β receptor 1 blocker) or PP2 (Src inhibitor). Scale bar = 50 μm. **(B)** Wound-healing assay in NPC cells with TGF-β and SB431542 or PP2. Left panel, representative images. Right panel, quantitative data. **(C)** Transwell assay for NPC cells treating with TGF-β and SB431542 or PP2. **(D)** Representative Western blots and the semi-quantitative assessments of the EMT marker (E-cadherin, vimentin and β-catenin) and total as well as Tyr416 phosphorylated Src expressions in NPC cells treated with TGF-β and SB431542 or PP2. # *P* < 0.01, + *P* < 0.001.

Since TGF-β-induced EMT has little been studied in NPC, we firstly wanted to confirm whether TGF-β stimulation promotes EMT in NPC cells. Hence, TGF-β was added into NPC cells. In CNE-1 cells, we noticed that TGF-β facilitates cell scattering with spindle-shaped morphological changes, while blocking TGF-β receptor 1 using SB431542 or suppressing Src Tyr416 phosphorylation using PP2 reversed the effect (Figure 3A). In the wound-healing assay, NPC cell migration was also significantly enhanced by TGF-β stimulation but was abrogated with additional SB431542 or PP2 (Figure 3B). Likewise, NPC cell motility was enhanced by TGF-β stimulation and suppressed by TGF-β receptor blocking or Src inactivation in the transwell assay (Figure 3C). Then, we wanted to confirm whether EMT or Src activity was promoted by TGF-β. Western blot was performed to evaluate the protein expressions. TGF-β stimulation significantly decreased the expression of E-cadherin (an epithelial phenotype marker), and increased vimentin (a mesenchymal phenotype marker) and β-catenin (a mesenchymal signal transducer) expression. When SB431542 or PP2 was added in, the alterations were suppressed (Figure 3D). These suggested that TGF-β initiates EMT via Src activation in NPC cells.

### TGF-β promotes EMT in NPC cells without affecting Flot2 expression

To further confirm that TGF-β promotes EMT in a dose dependent way, both 10 ng/ml and 20 ng/ml concentrations of the recombinant protein was added into both CNE-1 and 6–10B NPC cells.

**Figure 4:**
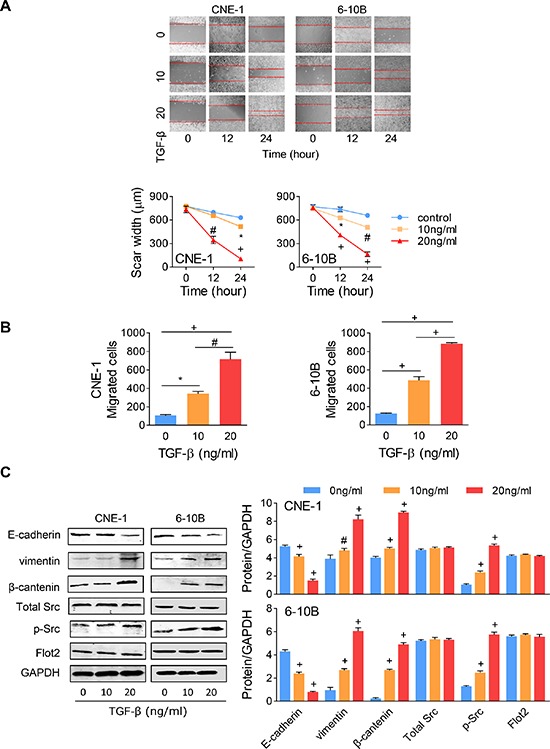
TGF-β-induced EMT has no influence on Flot2 expression **(A)** Wound-healing assay and **(B)** Transwell assay of NPC cells with different concentrations of TGF-β. **(C)** In Western blots assay, the EMT marker expressions (E-cadherin, vimentin and β-catenin) and Src phosphorylation were altered by TGF-β in a dose dependent way, but total Src expression and Flot2 expression was not affected by TGF-β. *P < 0.05, #*P* < 0.01, + *P* < 0.001.

After 24 hours, the scar area was significantly decreased at 10 ng/ml concentration and even completely healed at 20 ng/ml (Figure 4A). Under TGF-β stimulation, cell motility was significantly increased by more than 1 fold at the concentration of 10 ng/ml and dramatically increased to almost 7 folds at 20 ng/ml (Figure 4B). In protein level, as anticipated, Src phosphorylation was also enhanced by TGF-β does dependently, while total Src expression was not influenced under the same stimulation (Figure 4C). As to the EMT markers, along with TGF-β concentration increasing, E-cadherin expression was decreased, while vimentin and β-catenin expression was increased progressively. Then we are curious whether TGF-β stimulation increased Flot2 expression. To our surprise, no significant changes were found on the Flot2 expressions under either 10 or 20 ng/ml TGF-β stimulation (Figure 4C).

### Flot2 alone is not sufficient to affect NPC cell motility

Since Flot2 elevation was proved not the result of TGF-β-induced EMT, we then speculated whether Flot2 affect NPC cell metastasis. We used three siRNA sequences (siFlot2) to inhibit Flot2 expression in NPC cells.

**Figure 5:**
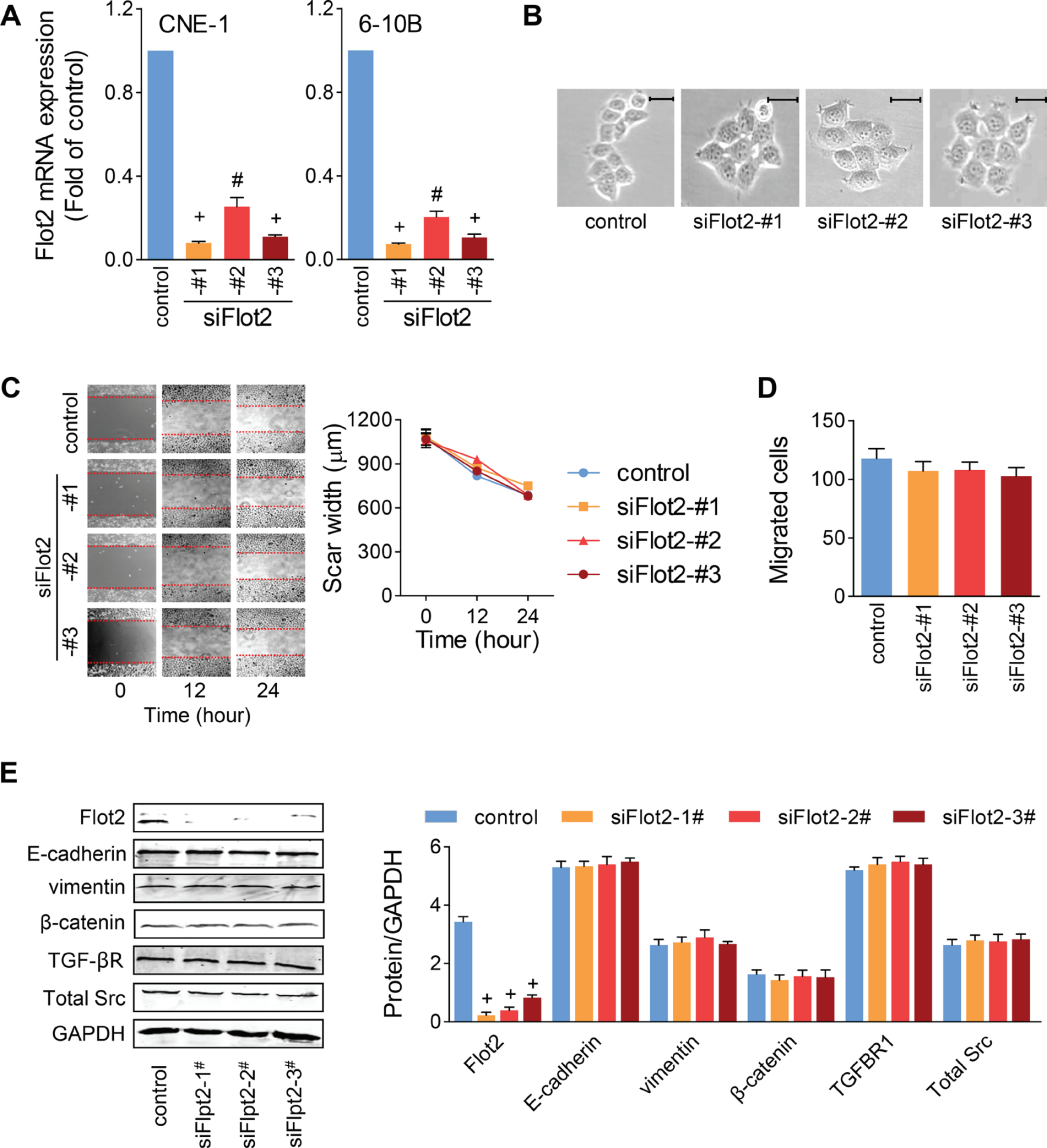
Silencing Flot2 alone does not changed NPC cell motility **(A)** Efficiency of three siRNA sequences against Flot2 was evaluated by Quantitative real-time PCR. **(B to D)** CNE-1 cell motility was not affected by any of the three Flot2-silencing siRNAs. (B) Cell scattering and morphology. Scale bar = 50μm. (C) Wound-healing assay and (D) Transwell assay. **(E)** Flot2 silencing has no influence on the expression of EMT makers (E-cadherin, vimentin and β-catenin), TGF-β receptor 1 or total Src in CNE-1 cells. #P<0.01, +P<0.001.

The efficiency of silencing was verified using Quantitative real-time PCR. All of the three siFlot2 successfully suppressed Flot2 mRNA expression, and siFlot2-#1 was the most efficient one (Figure 5A). These siFlot2 were added into CNE-1 and 6–10B cells. Out off our anticipation, silencing Flot2 did not alter cell morphology or cell scattering (Figure 5B). Likewise, neither in the experiment of transwell (Figure 5C) nor in the wound-healing assay (Figure 5D), none of the three Flot2 silencing sequences showed significant changes on the motility of NPC cells. In protein level, although all of the three siRNA sequences decreased Flot2 expression profoundly, it seemed they did not have significant influence on the expression of E-cadherin, vimentin or β-catenin (Figure 5E). We also hypothesized that Flot2 might be associated with TGF-β signaling. However, by Flot2 suppression, no alterations were found in the expression of the TGF-β receptor or Src (Figure 5E). These results indicate that altering Flot2 is not sufficient to influence NPC cell motility.

By this step, the laboratory experiments still could not answer our clinical observations why Flot2 was upregulated in the metastatic cases. It seems that Flot2 was neither the addition product of metastasis nor sufficient to induce metastasis, then we thought of another possibility: was it necessary to influence metastasis?

### Flot2 is necessary for TGF-β signal transduction in NPC cell metastasis

**Figure 6:**
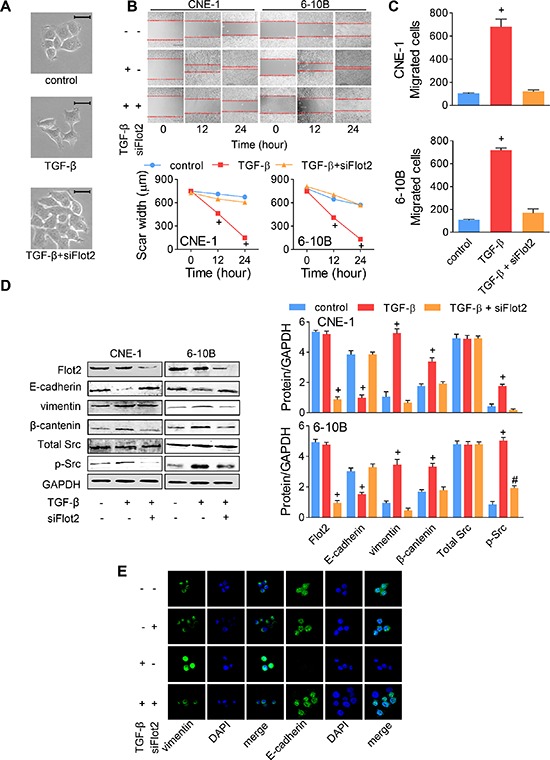
TGF-β-induced EMT was reversed by Flot2 silencing **(A)** TGF-β-induced CNE-1 NPC cell scattering and morphologic changes were reversed by siFlot2. Scale bar = 50 μm. **(B)** Wound-healing assay and **(C)** Transwell assay of NPC cells treated with TGF-β or siFlot2. **(D)** TGF-β-induced EMT maker (E-cadherin, vimentin and β-catenin) alterations and Src phosphorylation was reversed by siFlot2. **(E)** Immunofluorescence staining showed the E-cadherin and vimentin expressions changed by TGF-β and siFlot2 in CNE-1 cells. # *P* < 0.01, + *P* < 0.001.

It was recently reported that Flot2 kept Src activation in the rat optic nerve [20]. Hence, we then asked whether Flot2 was required in TGF-β signaling in human NPC cells. When siFlot2-#1 was added into the TGF-β cultured CNE-1 cells, cell scattering was dramatically suppressed, and cells were kept in colonic growth as the control and siFlot2-#1 groups (Figure 6A). We also noticed that TGF-β-induced pseudopod protrusions were retrograded by siFlot2 (Figure 6A). In the wound-healing assay, despite the rapid healing of the scar area promoted by TGF-β, cell migratory rate was significantly suppressed by Flot2 silencing (Figure 6B). In the transwell assay, silencing Flot2 under TGF-β stimulation suppressed it to the baseline level (Figure 6C). These suggested that Flot2 was essential for TGF-β-induced NPC cell metastasis. Then, we wanted to confirm the protein changes via western blot and immunofluorescence staining. In western blot, silencing Flot2 under TGF-β stimulation significantly inhibited Src phosphorylation, yet total Src expression was not influenced. As a result, E-cadherin, vimentin and β-catenin remained at the baseline level that similar to the TGF-β free control of CNE-1 and 6–10B cells (Figure 6D). As shown under the confocal fluorescence microscope, TGF-β stimulation decreased E-cadherin expression and increased vimentin expression in CNE-1 cells, while silencing Flot2 kept E-cadherin and suppressed vimentin expression under TGF-β stimulation. No obvious difference was found between the group of control and silencing Flot2 alone (Figure 6E). Finally, we conclude from these results that Flot2 was, though not sufficient, yet necessary and essential for the TGF-β-induced EMT signal transduction.

## DISCUSSION

In this study, we firstly verified that Flot2 was a pathological NPC biomarker for lymphatic and distant metastasis. As a consequence, Flot2 elevation predicts poor prognosis of high recurrence and mortality for NPC patients. Accordingly, we wanted to figure out the underlying mechanisms. We proved that Flot2 alone was not sufficient to influence NPC cell metastasis. Flot2 elevation was not the consequence of TGF-β stimulation, but TGF-β could induce EMT in NPC cells via Src activation. Finally, we gave evidence that Flot2 was, though insufficient, yet necessary for TGF-β signaling and Src activation in the EMT process of NPC cells.

NPC is an uncommon epithelial cancer worldwide, but has high rate of occurrence and subsequence metastasis among people in southeastern China, especially in Cantonese region [12]. It is known that EMT is one of the most important manners for cancer metastasis, and TGF-β was proved to boost EMT [1–6, 8]. Here, we gave evidence that TGF-β stimulation induced EMT in NPC cells via Src activation. By blocking TGF-β signaling or inhibiting Src activity using the specific inhibitors, EMT was suppressed. This proved that Src is a key regulator of TGF-β-induced EMT in NPC cells. Previously, TGF-β-induced EMT process has been proven to be Src-dependent in breast cancer [2]and pancreatic cancer [1], but not in NPC yet. To our knowledge, this is the first study demonstrating that EMT was enhanced by TGF-β stimulation and subsequent Src phosphorylation in NPC. Although elevated Flot2 was showed to be correlated with NPC metastasis by our clinical data, our laboratory experiment showed that Flot2 level was not influenced by TGF-β stimulation. This suggested that Flot2 elevation was independent of TGF-β stimulation. In other words, Flot2 elevation is not the compensative result of EMT or cancer metastasis.

Previous studies have shown that Flot2 act as a stabilizer of some membrane-associated proteins to keep their functions [13]. In contract with previous studies that silencing Flot2 could suppress the metastatic capacity of some cancer cells [18, 21], our experiments demonstrated that simply silencing Flot2 without any other stimuli was not sufficient to change either cell motility or EMT marker expressions. This also suggested that Flot2 is not the stabilizer of E-cadherin or TGF-β receptor in NPC. However, under TGF-β stimulation, things came quite different. We proved that silencing Flot2 suppressed TGF-β-induced EMT by inhibiting Src phosphorylation. These data not only demonstrate that Flot2 is necessary for NPC metastasis, but also reveal that Flot2 play as a key node of TGF-β-induced EMT. Given the divergence of Flot2 in NPC and other cancers, we speculated that metastatic regulating network is not complete the same in all cancers, and NPC Flot2 may be a special case. On the other hand, it was also reported of many exceptions that TGF-β alone was not sufficient to induced EMT in many cancer cell lines [22], which requires some other downstream signaling activation [6]. Hence, taken together, the laboratory experiments answered our clinical observations that Flot2 is necessary, though not sufficient, to cooperate with Src phosphorylation in the TGF-β-induced NPC metastasis.

**Figure 7:**
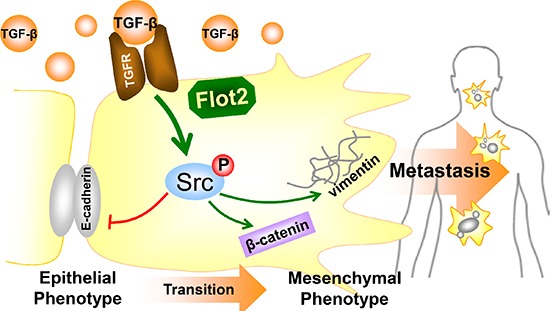
Summarizing diagram of this study Summarizing diagram of this study. Flot2 is indispensable for Src phosphorylation in TGF-β signaling. Silencing Flot2 reverses the TGF-β-induced EMT in NPC cells by suppressing Src activation. By this, E-cadherin expression is upregulated and vimentin and β-catenin expressions are downregulated. These findings answer our clinical observations why Flot2 elevation is associated with more lymphatic as well as distant metastasis, and consequently lead to adverse survival outcomes.

In conclusion, we discovered the clinical significance of Flot2 in promoting NPC metastasis and consequently leading to adverse prognosis. Then, our experiment showed that TGF-β-induced Src activation and promotes EMT in NPC cells. In cooperation with Src phosphorylation, Flot2 was an indispensable member for TGF-β signaling. Taken these together, our data suggest that suppressing Flot2 may be a novel way against TGF-β-induced EMT in NPC (Figure 7).

## MATERIALS AND METHODS

### Patients and tumor tissue samples

This study was approved by the Ethics Review Broad of Nanfang Hospital, and tissues were obtained with the consent of each patient. Paraffin-embedded pathological specimens were from 181 NPC patients that received NPC resection surgery or electronic epipharyngoscope biopsy between 2004 and 2008. All the patients were diagnosed according to the UICC/AJCC 1997 staging system of NPC. None of the patients received any anticancer therapy before the specimen was obtained.

### Immunohistochemistry staining

Immunohistochemistry staining was performed according to the standard protocol as previous described [23]. Polyclonal rabbit primary antibody for Flot2 (Abcam, San Francisco, CA), HRP-conjugated secondary antibody and DAB staining kit (CWBIO, Beijing, China) were used in the experiment. The intensity of staining was scored as 0 (negative), 1 (weak), 2 (medium) or 3 (strong), while the extent of staining was scored as 0 (0% of cell stained), 1 (1–25%), 2 (26–50%), 3 (51–75%) or 4 (76–100%). Then the intensity and the extent scores were multiplied as the final staining scores (0–12) for Flot2 expression. Tumors of final staining score 0–2 were considered as negative, 3–5 as low expression, and 6–12 as high expression.

### Cell culture

NPC cell lines of CNE-1 and 6–10B were cultured in RPMI 1640 medium with L-glutamine (Hyclone, Logan, UT, USA) and 10% fetal bovine serum at 37°C under 5% CO_2_. SiRNA transfection was carried out 72 hours before the experiment using the Lipofectamine^®^ 2000 Transfection Reagent from Invitrogen (NY, USA) following the manufacturer's recommended protocol. The siRNA targeted sequences of Flot2 were as follows: siFlot2-#1 GCAGAGAGATGCTGACATT, siFlot2-#2 CCAAGATTGCTGACTCTAA and siFlot2-#3 GCAGG AAGAGATTGAGATT. For TGF-β stimulation, cells were cultured with the medium with 10ng/ml or 20ng/ml recombinant human TGF-β (Abnova, Taipei, China) for 6 day before the experiments. Src tyrosine kinase inhibitor PP2 (Sigma-Aldrich, St. Louis, MO, USA) or TGF-β receptor 1 inhibitor SB431542 (Santa cruz, Dallas, TX, USA) were added into the medium when intended.

### Wound-healing assay

Cells were seeded on a 12-well plate at a density of 5 × 10^4^ per well and incubated overnight. Cells were scratched using a 200 μl plastic filter tip. Wound closure was observed at 0, 12 and 24 hours under an inverted microscope.

### Transwell assay

Transwell assay was performed using Boyden's chamber, and the upper chamber were filled with fetal bovine serum free RPMI 1640 medium while the lower chamber were with 30% fetal bovine serum. After transfer into upper chamber at 2 × 10^4^ cells per well, cells were incubated at 37°C for 48 hours. The cells that invaded through the membrane were fixed in 4% paraformaldehyde for 20 min and stained with 0.1% crystal violet for 30 min. The results were observed under inverted microscope.

### Quantitative real-time PCR

The total NPC cells RNA was extracted using Trizol Kit (Takara Bio Inc., Shiga, Japan) following the manufacturer's protocols. cDNA was synthesized using the First Strand cDNA synthesis Kit (Takara). Quantitative PCR was performed using the SYBR Green dye (Roche, Mannheim, Germany). The primers were as followed: FLOT2 (5′-GAGATTGAGATTGAGGTTGTG-3′ and 5′-ATCCCCGTATTTCTGGTAGG-3′) and GAPDH (5′-ACTTCAACAGCGACACCCACTC-3′ and 5′-TACCA GGAAATGAGCTTGACAAAG-3′).

### Western blotting

Total protein were extracted and subjected to Western blotting as previous described [23]. Monoclonal rabbit primary antibodies against E-cadherin, β-catenin, vimentin, Src and p-Src (Tyr416) (Cell Signal Technology, Boston, MA, USA), polyclonal rabbit primary antibody against Flot2 (Abcam, San Francisco, CA, USA), TGF-β RI (Santa Cruz, Dallas, TX, USA) and the secondary fluorescence goat anti-rabbit antibody (LI-COR, Lincoln, NE, USA) were used in this experiment. The blots were scanned using Odyssey imaging system (LI-COR).

### Immunofluorescence staining

After fixed in 4% paraformaldehyde for 20 min and permeabilized in 1% Triton dilution for another 20 min, cells were blocked by 3% BSA dilution. Primary antibodies against E-cadherin and vimentin (Cell Signal Technology) were used according to the recommended protocols and incubated overnight at 4°C. Then cells were incubated with secondary antibodies (Beyotime, Shanghai, China) for 1 h at room temperature. The results were observed under Olympus FluoView confocal microscope (Olympus Optical, Tokyo, Japan).

### Statistical analysis

*P*-value less than 0.05 was considered as statistically significant and determined by Student's *t*-test as indicated in figure legends. One-way ANOVA analysis was performed to evaluate the statistical significance among multiple variables. Spearman correlation coefficients were calculated between the differential classification and Flot2 score. Cox regression model was used to find out the independent prognostic factors. The event was defined as a cancer-related death. Kaplan-Meier curves were drawn to present overall survival differences between categorized groups, and the significance was evaluated by Chi-square test.
